# Mild cognitive impairment in the elderly Relationship between communication and functional capacity

**DOI:** 10.1590/1980-57642018dn12-020009

**Published:** 2018

**Authors:** Ana Iza Gomes da Penha Sobral, Cláudia Marina Tavares de Araújo, Marcos Felipe Falcão Sobral

**Affiliations:** 1 PhD Student in Cognitive Psychology at the Federal University of Pernambuco, Recife, PE, Brazil.; 2 Speech Therapist. Assistant Professor at the Phonoaudiology Department, Federal University of Pernambuco, Recife, PE, Brazil.; 3 Assistant Professor at the Federal Rural University of Pernambuco, Recife, PE, Brazil.

**Keywords:** mild cognitive impairment, elderly, communication, comprometimento cognitivo leve, idoso, comunicação

## Abstract

Mild Cognitive Impairment is characterized as an intermediate form between age-related change and dementia. For the elderly, autonomy and independence are related to the ability to remain active in conducting their social activities and, for this to occur, communication is fundamental in this process.

**Objective:**

To assess the association between communication and the abilities of elderly people with mild cognitive impairment to perform instrumental activities of daily living.

**Methods:**

A cross-sectional, quantitative, analytical, correlational study was conducted at the Open University of the Third Age (UnATI), a program of the Federal University of Pernambuco. This study included 92 people, comprising 46 elderly with mild cognitive impairment and a caregiver or family member who met the inclusion criteria. The elderly were asked to complete a sociodemographic questionnaire and Lawton-Brody’s Instrumental Activities of Daily Living Scale. The caregivers were asked to complete the Functional Assessment of Communication Skills. The following variables were studied: social communication skills and instrumental activities of daily living. Data were stored in an Excel^®^ 2007 spreadsheet, and the Pearson correlation test was used for the statistical analysis.

**Results:**

There were statistically significant correlations in four domains of social communication: referring to family members by name (p=0.0033); requesting information about people or events (p=0.0355); understanding conversations in a noisy environment (p=0.0448); and understanding what they watch on television or listen to on the radio (p=0.0127).

**Conclusion:**

Changes in the communication of elderly people with mild cognitive impairment interfere with their ability to perform instrumental activities autonomously and independently.

## Resumo

O comprometimento cognitivo leve se caracteriza como forma intermediária entre a alteração ocasionada pela idade e a demência. Para o idoso, autonomia e independência estão relacionadas à capacidade de se manter ativo na realização das suas atividades sociais e, para que isso aconteça, a comunicação se torna fundamental neste processo.

**Objetivo:**

Avaliar a relação entre comunicação e desempenho dos idosos com comprometimento cognitivo leve na realização das atividades instrumentais da vida diária.

**Métodos:**

Estudo transversal, quantitativo, analítico e correlacional, realizado na Universidade aberta à Terceira Idade da Universidade Federal de Pernambuco. Participaram do estudo 92 pessoas, sendo 46 idosos com comprometimento cognitivo leve e seus respectivos cuidador ou familiar que atenderam aos critérios de inclusão. Aos idosos, foram aplicados o questionário sociodemográfico e a Escala de Lawton-Brody; e, a seus informantes, a escala de Avaliação Funcional das Habilidades Comunicativas. As seguintes variáveis foram estudadas: habilidades de comunicação social e atividades instrumentais de vida diária. Os dados foram organizados em planilha Excel^®^ 2007 e para análise estatística foi aplicado o Teste de Correlação de Pearson.

**Resultados:**

Evidenciou-se correlação estatisticamente significativa em quatro domínios da comunicação social, a saber: nomeia pessoas familiares pelo nome (p=0,0033); solicita informação sobre pessoas ou acontecimentos (p=0,0355); compreende conversas em ambiente barulhento (p=0,0448) e, compreende o que assiste na televisão ou ouve no rádio (p=0,0127).

**Conclusão:**

as alterações existentes na comunicação de idosos com comprometimento cognitivo leve interferem na capacidade de realização de atividades instrumentais com autonomia e independência.

Faced with the current demographic changes that have led to a population with an increasing life expectancy, the importance of guaranteeing the elderly not only greater survival but also a better quality of life is evident.^1^

For the elderly, quality of life is associated with the ability to remain autonomous and independent in the performance of daily activities, especially with regard to social interactions with family, friends, and neighbors.^2^

Activities of daily living (ADL) can be classified into Basic Activities of Daily Living (BADL), related to tasks of self-care, such as food preparation and consumption, personal hygiene and dressing, and into Instrumental Activities of Daily Living (IADL) that include activities which require more complex cognitive abilities in terms of neuropsychological requirements and the influence of social, motivational, and contextual factors for the maintenance of independent life; for example, going somewhere, administering medication, and using the telephone.^3^ Instrumental activities are commonly used in studies aimed at identifying the independence and autonomy of the elderly.^4^^,^ 5 One of the ways to evaluate ADL is by the Lawton and Brody scale (1969), an easy-to-apply tool used worldwide. Social engagement occurs primarily through one of the cognitive skills, communication.^6^ Commonly, the aging process causes changes in communication,^7^^,^ 8 whether due to disorder or impairment in neurological processes or the decreased mobility and muscular strength of the phonoarticulatory organs.^8^

Oral language is the most widely used form of communication. It has its own characteristics and changes in the different phases of life. With advancing age, it tends to become less efficient, causing changes in dialog or communicative cycles^8^ and, consequently, in the social relationships of the elderly. Communication may be an important indicator of the cognitive capacity of the elderly, reflecting preserved or altered aspects.^6^

The increase in life expectancy has promoted interest in aspects involving cognition. In a systematic review on the communication of the elderly with MCI, it was demonstrated that elderly people have difficulties in expressive and receptive communication.^9^

Diseases that compromise cognition have been increasingly studied in order to understand the differential threshold between senescence and senility.^10^ Concerned with the increasing number of people with cognitive impairment, the American Academy of Neurology recommended diagnosis and follow-up of elderly people with mild cognitive impairment (MCI).^10^ This condition consists of cognitive loss compared to people of the same age group but does not meet the criteria for dementia.

Laks et al.^11^ affirmed a link between cognitive performance and functional impairment in ADL, thus being important for investigation and intervention in this population. Elderly with MCI may gradually lose their capacity for occupational performance, and their social relationships may be affected. Thus, the evaluation of cognitive functions can detect this condition early, enabling the elderly and their families to take measures that can prevent or delay the social and emotional impact that may result from the development of dementia.^12^

Since the completion of IADL involves communication and that language is an instrument of socialization, which is a mediator in the relationship between humans and their world, it is important to know the aspects of this relationship so that a plan of action can be devised that integrates activities of health promotion, prevention, and treatment focused on a better quality of life for the elderly.^13^ Given this panorama, the present study aims to present the relationship between social communication and performance, as assessed by IADL, in elderly with MCI. Therefore, the hypothesis of this study is that elderly with MCI exhibit changes in social functions and in the performance of instrumental activities.

## METHODS

### Ethical aspects

This study was approved by the Human Research Ethics Committee of the Federal University of Pernambuco (UFPE) under protocol CAAE No. 26145813.8.0000. 5208.

Participants were asked to sign the Free and Informed Consent Form, after due clarification concerning the study and before data collection.

### Casuistic

A cross-sectional, analytical, correlational study was carried out at the Open University of the Third Age (UnATI) of the Federal University of Pernambuco from May to September 2014. The study was conducted with 46 elderly people enrolled in the UnATI and their respective caregivers and/or family members, giving a total of 92 persons interviewed.

### Inclusion and exclusion criteria

Elderly people duly enrolled in the UnATI were selected for this study. Participants were submitted to the diagnostic criteria for MCI of Petersen (2001): [a] self-report of memory impairment corroborated, if possible, by a close informant; [b] not meeting the criteria for the diagnosis of dementia based on an objective measure of affected components; [c] preservation of the instrumental activities of daily living; and [d] cognitive impairment, as evidenced by specific tests. Cognitive impairment was self-reported by the elderly and confirmed by the objective cognitive tests.

It is important to note that this classification was performed, taking into account the criteria described above, with no psychiatric and/or neurological evaluation. The elderly participants presented an objective cognitive impairment, as measured by the Mini-Mental State Examination (MMSE), the Clock Drawing test, and the animal category of the verbal fluency test. In addition, they scored between 0 and 5 points on Yesavage’s Geriatric Depression Scale (GDS-15), which indicates an absence of depressive signs and symptoms.

Elderly who were illiterate, lived alone, had neurological, psychiatric, orthopedic or rheumatologic diseases, or any other motor or sensory limitations that could compromise the performance of IADL were excluded from the study.

### Instruments

*Sample selection.* For sample selection, the following tests were applied:

1. Mini-Mental State Examination (MMSE): Employed to screen for the presence or absence of cognitive impairment.^13^ The MMSE is an instrument for cognitive screening validated for use in Brazil. The instrument has cut-offs based on level of education of the participant. It is one of the most used cognitive screening instruments worldwide.^14^
2. Geriatric Depression Scale (GDS): Measures the symptoms of depression. In this study, the short form was used with 15 questions identifying the symptoms of depression and related risk factors.^15^
3. Semantic verbal fluency test - animals category: The test entails asking the person to recite as many names of animals as he or she can remember in one minute.^16^ This test was used because it is believed that the identification of animals would be common to all people enrolled, regardless of their level of education.
4. Clock Drawing Test (CDT): An evaluation tool used for cognitive screening.^17^It evaluates several cognitive dimensions, such as memory, visual motor functions, executive function and verbal comprehension. The score ranges from 0 to 5 points, based on the scoring table of Shulman et al. (1993), a 5-point scale with a cut-off point of 3.

*Data collection.* The elderly completed the following instruments:

1. Sociodemographic and clinical questionnaire: Including information on participant name, age, sex, marital status, children, with whom they live, education, professional training, functional data, religion, diagnosed comorbidities, and leisure activities, among others.
2. Lawton-Brody’s IADL (1969): Adapted to the Brazilian context by Santos and Junior in 2008,^16^ this scale evaluates IADL.
3. The ASHA FACS (Functional Assessment of Communication Skills for Adults - American Speech-Language-Hearing Association) communicative skills scale, which consists of an instrument for the evaluation of communicative abilities,^18^ was applied to the family member and/or the responsible caregiver. For the purpose of this study, the category of social communication, which constitutes the variable of interest, was used for the correlational analysis. In this study, the 21 questions of the Social Communication Questionnaire were applied.

### Procedures for data collection

Initially, the elderly who were enrolled in the UnATI were screened based on their self-reports of cognitive alterations over the last year. Individuals who met the inclusion criteria were invited to participate in the study and a day and time were scheduled for the data collection. Interviews took place in an appropriate environment, the UnATI room, and lasted approximately 30 minutes. The elderly were selected based on the cut-off points established by each instrument. Many of those initially selected did not meet the previously established criteria and were subsequently excluded from the study.

Those eligible to participate in the survey answered the sociodemographic and clinical questionnaire. After this procedure, the elderly person was asked to leave the room, and their caregiver/family member was called in. The ASHA FACS communication skills scale was then administered to the caregiver/family member, with an average duration of 20 minutes.

### Data analysis

The statistical analysis software IBM SPSS Statistics 19.0 was used for the descriptive measures of numerical and ordinal variables: mean, median, mode, standard deviation, maximum value, minimum value and quartiles, as presented in Table 1. Subsequent to the presentation of these results, tables were generated with the absolute, relative, and accumulated values of the variables to verify outliers and generate distribution charts to verify the behavior of the variables to be analyzed.

**Table 1:** Characteristics of screening test performance.

	Lawton-Brody Scale				
	N	MMSE	GDS-15	CDT	VF
Mean	46	22.39	–	–	–
Median	46	23	–	–	–
Mode	46	23	1	2	9
Standard dev.	46	1.422	–	–	–
Minimum	46	19	1	1	5
Maximum	46	27	2	2	9
Quartile 25^th^	46	21.75	–	–	–
Quartile 50^th^	46	23	–	–	–
Quartile 75^th^	46	23	–	–	–

MMSE: Mini-Mental State Examination; GDS-15: Short Yesavage Geriatric Depression Scale (GDS-15); CDT: Clock Drawing Test; VF: Verbal Fluency.

In order to study the correlation between the variables as described in the study objectives, variables for totals on the Lawton-Brody scale and the ASHA FACS scale were created. The variables were tested with those described using the Pearson’s correlation test, considering a positive or negative correlation between the variables tested when the p-value of the respective test was less than 5% (p<0.05).

## RESULTS

Of the 46 elderly study participants, 45 (97.8%) were female. The mean age was 70.3 years (standard deviation ±6.663). Regarding marital status, the percentage of married and widowed elderly was the same at 39.1% respectively, followed by 17.4% divorced and 4.3% unmarried. The average number of children was 3.35 (s.d. ±2.213).

For 50.0% (n=23) of the elderly, the main source of income was retirement, followed by 23.9% (n=11) pensioners; 21.7% (n=10) were dependent on their husbands, and the remaining percentage, 4.4% (n=2), reported obtaining support through various forms, including help or small commercial activities. The predominant occupation was that of housewife 21.7% (n=10), with the remainder having a variety of different jobs, such as salesperson, seamstress, teacher, and secretary.

The most frequent comorbidity was arterial hypertension, which affected 43.5% (n=20) of the study population. Regarding the main leisure activity of the elderly, 58.7% (n=27) reported watching television, 30.4% (n=14) preferred reading as entertainment, followed by 8.7% (n=4) who preferred talking with family or friends, and 2.2% (n=1) stated no leisure activity.

The mean score obtained on the MMSE was 22.39 points, and the median was 23 points. The mode on the verbal fluency test was nine. Table 1 shows the mean, median, mode, maximum, minimum, quartiles, and standard deviations of the instruments used in selecting the elderly to participate in this study.

For the ADL, 80.4% (n=37) of the elderly were categorized into the partially dependent condition. The activities that presented the greatest difficulty were: “use of medications” (63%, n=29) and “domestic work” (58.7%, n=27). Simultaneously, “purchasing,” “meal preparation,” and “travel” showed equal incidence (19.6%, n=9). The highest degree of independence was observed for “telephone use” (93.5%, n=43), followed by “money handling” (84.8%, n=39).

In this study, a statistically positive relationship was found for four areas in a correlation between the social domain of the ASHA FACS scale and level of independence, as evaluated by the Lawton-Brody scale, including requests information about people or events (p=0.0355) and understands what he or she watches on television or listens to on the radio (p=0.0127).

The ASHA FACS social communication variables that demonstrated a significant relationship are presented in Table 2. Four of the twenty-one questions in the field of Social Communication were significantly related to the ASHA FACS scale, as shown in Table 2. The values of Pearson’s correlation shown measure the relationship between the skills studied and the Lawton-Brody Scale scores.

**Table 2:** Relationship between social communication components of the ASHA FACS scale and performance on Lawton-Brody scale.

Question	Lawton-Brody Scale					
	N	%	Mean	Standard deviation	Pearson correlation	P-value
Refers to family members by name	2	4	15.500	0.707	0.4246	0.0033
	44	96	19.091	1.611		
Requests information about people or events	5	11	17.400	2.074	0.3108	0.0355
	41	89	19.122	1.631		
Understands conversations in noisy environments	1	2	17.000	–	0.2973	0.0448
	30	65	18.667	1.788		
	15	33	19.600	1.2		
Understands what they watch on TV or listen to on the radio	27	59	18.407	1.803	0.3645	0.0127
	19	41	19.684	1.376		

## DISCUSSION

Communication is one of the main cognitive components necessary for an individual to perform instrumental activities of daily living and social interaction autonomously and independently. In this study, we identified the relationship between the main components of social communication that are significantly related to performance in more complex instrumental daily functions.

Regarding the demographic characteristics, there was a predominance of females in the sample, as is the case with most studies involving elderly populations.^19^ The fact that elderly women featured as a majority may be associated with their higher life expectancy resulting from, among other factors, differences in lifestyle and attitudes towards diseases and disabilities.^20^

Women are typically more concerned with self-care and health. They show interest in participating in activities, seeking new acquaintances, and expanding their circles of friendships.^21^ On top of this is the assumption that older people participate less in collective actions for sociocultural reasons.^22^ Similar findings were found in another study, whose percentage of males did not exceed 10.0%.^23^

In other studies, the mean age of the elderly participants was 70.3 years.^24^^,^ 25 It is assumed that the low participation of older people aged between 75 and 84 years may be related to the degree of dependence and comorbidities associated with aging, thus constituting factors limiting access to and participation in social activities.^21^

Regarding marital status, there was an equal proportion of married and widowed elderly (39.1%). This corroborates data found by a study carried out in Goiana City, where 45.3% of the elderly were married and 44.0% widowed.^25^ One of the predisposing factors for an increase in the number of widows is of sociocultural origin, since women tend to marry older men. Moreover, it is associated with a higher rate of male mortality and the fact that men, when widowed, often remarry.^23^ Nevertheless, a survey conducted in 11 European countries showed that about 70.0% of the elderly assessed were married.^26^ However, in Brazil, studies showed a predominance of widowhood among the elderly, especially females.^27^^,^ 28

Currently, the role of the elderly is usually restricted to the domestic environment. The task of running the home and taking care of the children is practically their responsibility. This fact is reflected in the findings of the present study, since most of the women interviewed were not engaged in professional activities. This finding is in line with other surveys.^29^^,^ 30

Empirical studies involving the elderly population report hypertension as the most frequent comorbidity.^23^^,^ 29 Results of the present study corroborated these studies; however, it is worth noting some factors that contributed to this result: the large number of elderly women participants, and the fact that women are more aware of diseases and have a greater tendency toward self-care, in addition to attending health services more than the male population.

Although participants claimed to have time available to perform various leisure activities, the elderly in this study reported watching television as their main form of entertainment. This is similar to results found in several studies available in the current literature, such as the study conducted in São Paulo involving participants in community groups,^31^ and others that describe leisure as one of the indicators of social life for the elderly population.^32^^,^ 33

Several factors may explain the findings described above, such as socioeconomic conditions, education, and culture and lifestyle, as well as the physical and/or psychological limitations that often restrict the elderly to their home environments. In the present study, the authors believe that although many of the elderly were independent, it was observed that the use of television demonstrates an idle, passive form of leisure, performed only as a way of occupying time. Added to this is the fact that watching television is associated with a lack of company, where elderly may use television to fill the gap left by children and grandchildren as they leave home.

In senescence, communication plays a central role in the subject’s relations with the world, favoring their active participation in different environments, and constitutes a decisive factor for independence, autonomy, well-being, and happiness. Studies have documented that high rates of social activities are associated with improved functioning,^34^^,^ 35 greater independence for IADL, and better social communication. Participation in social activities stimulates several cognitive functions, including the communicative process, which can be defined as a way of exchanging experiences,^35^^,^ 36 mainly through oral language.

The investigation into the association between social relations, language, and cognition in older Americans demonstrated that greater engagement in social activities was related to better language skills.^37^ The findings obtained in the present study corroborate the results of the study mentioned above.

The communication abilities of the elderly with MCI may be altered at the first signs of impairment.^38^^,^ 39 In the scientific literature, the communication complaints of elderly people with MCI are rarely explicit and are frequently associated with memory impairment, such as difficulties in naming and remembering words.^9^ The compensatory communicative capacities are evidenced when applying a questionnaire on communication, and the result demonstrates the need for independence in daily life communication.^14^

The ability of the elderly to perform a naming task was investigated in a study involving participants without cognitive impairment, and those with early and moderate Alzheimer’s disease. The results showed that there were no significant differences between the groups for naming, although an association was found with an increase in age.^40^ On the other hand, naming difficulties have been reported in the elderly with MCI,^9^ as well as problems with different components of semantic cognition, especially memory.^41^ These changes are due to failure in the executive process of semantic activation control; as the disease develops, semantic knowledge also begins to degrade.^42^

The ability of the elderly to produce and hold a discourse is important in the differentiation of functioning levels of individuals with MCI and those with dementia or without cognitive impairment, and this is also associated with the search for new information about people or events.^41^ In this study, the ability of the elderly to hold a discourse was assessed based on questions 16 to 19 of the ASHA FACS scale.

The present study identified that the elderly with MCI had a reduced capacity for this function. Their interest was more restricted to information about family members and shorter speech.

In search of information about the speech of people with MCI, Baek et al.^42^ conducted research by recalling history and inference by mirroring the cognitive demands of everyday speech, including conversation. The authors showed that this ability was impaired in the elderly with MCI, corroborated by the data found in our study.

The discourse of these elders contains semantic paraphasias, that is, the substitution of one word for another, and lexical access failures, defined as difficulties in recognizing and naming figures and symbols, decreasing access to information and hampering understanding of more complex subjects.^9^ Many of the caregivers or family members reported that the elder became confused when trying to name some commonplace objects, use the term “escapes my memory,” or end up replacing the terms with others that work, thus demonstrating their compensatory abilities.

With regard to questions involving understanding television or radio and conversations in noisy environments, data from this study matched that found in the existing literature. The present study indicates that this difficulty may be related to the loss of ability to perform temporal processing of sounds associated with the aging process.^43^ However, it is noteworthy that elderly people with MCI need more time to process the information received due to the gradual decline in cognitive functions. Furthermore, it is sometimes difficult to follow informative news. The slowing down of cognitive processing influences other functions, such as divided attention, which leads to more distractions for the elderly. This difficulty is one of the limiting factors in understanding conversations in noisy environments, which also interferes with the performance of social activities.

Several hypotheses have been proposed to justify the difficulty understanding speech in the elderly: auditory impairment and cognitive alteration. In most cases, these occur simultaneously.^44^ Although there were no reports of hearing difficulties, this ability was not formally evaluated.

In this study, there were no reports of hearing difficulties, and it was assumed that there was more cognitive alteration in this domain. In MCI, the relationships between cognition and hearing are still unclear. It is known that elderly people have a higher prevalence of hearing complaints, albeit secondary to presbycusis or hearing loss due to the aging process. These are all more severe when compared to control groups. Presbycusis contributes to the reduction of speech recognition, as it increases the difficulties of central auditory processing through auditory functions and the effects of background noise, which causes greater distraction of auditory attention.^45^ In this study, however, the elderly with MCI and their caregivers/relatives did not report complaints related to hearing difficulties.

Elderly patients with MCI exhibit changes in social communication that interfere with the performance of ADL, often leading to a decrease in autonomy and independence. Thus, these changes can be prevented or reduced if the elderly are guided by a competent professional, and a treatment program is initiated with the aim of improving the altered components.

One of the strategies that may benefit older people with MCI is cognitive training, since communication is part of the broad and complex network of cognition. Studies show that near-transfer training provides gains in functional aspects, such as carrying out everyday tasks and improving the autonomy and independence of the elderly.^46^^,^ 47

The results of this study demonstrate the importance of interventions in social communications among the elderly with MCI. Such intervention seeks to help maintain a more independent life, which may favor active aging with quality-of-life.

Limitations of this study include the fact that the elderly were not evaluated by a psychiatrist and/or neurologist, nor did they undergo a neuropsychological evaluation to obtain an accurate and detailed diagnosis. Only the MMSE was used and the number of elderly individuals who reported cognitive alterations but had normal performance on tests was high. Finally, reconciling the simultaneous collection of data from the elderly person and the caregiver/family member proved difficult. Therefore, future studies should use a battery of neuropsychological tests to confirm the diagnosis.

In conclusion, because it relates to multiple cognitive abilities, communication constitutes a necessary function for the socialization of individuals. Elderly patients with MCI have cognitive deficits that can cause greater communicative losses and, consequently, restrictions in their performance of daily activities. This study demonstrates the relationship between performance of IADL and social communication in the elderly with MCI for four of the 21 domains.

Four components had a significant association with IADL: referring to family members by name, requesting information about people or events, understanding conversations in noisy environments, understanding what they watch on TV or listen to on the radio.

It is important to emphasize that communication abilities in elderly with MCI are seldom explored in the literature. Thus, research in this area is necessary to obtain information that may help in understanding the complex relationships within the world of the elderly person.
